# Deep-learning algorithms for the interpretation of chest radiographs to aid in the triage of COVID-19 patients: A multicenter retrospective study

**DOI:** 10.1371/journal.pone.0242759

**Published:** 2020-11-24

**Authors:** Se Bum Jang, Suk Hee Lee, Dong Eun Lee, Sin-Youl Park, Jong Kun Kim, Jae Wan Cho, Jaekyung Cho, Ki Beom Kim, Byunggeon Park, Jongmin Park, Jae-Kwang Lim

**Affiliations:** 1 Department of Emergency Medicine, College of Medicine, Yeungnam University, Daegu, Korea; 2 Department of Emergency Medicine, Daegu Catholic University School of Medicine, Daegu, Korea; 3 Department of Emergency Medicine, Kyungpook National University, Daegu, Korea; 4 Department of Emergency Medicine, Daegu Fatima Hospital, Daegu, Korea; 5 Department of Radiology, Daegu Fatima Hospital, Daegu, Korea; 6 Department of Radiology, School of Medicine, Kyungpook National University, Daegu, Korea; University Magna Graecia of Catanzaro, ITALY

## Abstract

The recent medical applications of deep-learning (DL) algorithms have demonstrated their clinical efficacy in improving speed and accuracy of image interpretation. If the DL algorithm achieves a performance equivalent to that achieved by physicians in chest radiography (CR) diagnoses with Coronavirus disease 2019 (COVID-19) pneumonia, the automatic interpretation of the CR with DL algorithms can significantly reduce the burden on clinicians and radiologists in sudden surges of suspected COVID-19 patients. The aim of this study was to evaluate the efficacy of the DL algorithm for detecting COVID-19 pneumonia on CR compared with formal radiology reports. This is a retrospective study of adult patients that were diagnosed as positive COVID-19 cases based on the reverse transcription polymerase chain reaction among all the patients who were admitted to five emergency departments and one community treatment center in Korea from February 18, 2020 to May 1, 2020. The CR images were evaluated with a publicly available DL algorithm. For reference, CR images without chest computed tomography (CT) scans classified as positive for COVID-19 pneumonia were used given that the radiologist identified ground-glass opacity, consolidation, or other infiltration in retrospectively reviewed CR images. Patients with evidence of pneumonia on chest CT scans were also classified as COVID-19 pneumonia positive outcomes. The overall sensitivity and specificity of the DL algorithm for detecting COVID-19 pneumonia on CR were 95.6%, and 88.7%, respectively. The area under the curve value of the DL algorithm for the detection of COVID-19 with pneumonia was 0.921. The DL algorithm demonstrated a satisfactory diagnostic performance comparable with that of formal radiology reports in the CR-based diagnosis of pneumonia in COVID-19 patients. The DL algorithm may offer fast and reliable examinations that can facilitate patient screening and isolation decisions, which can reduce the medical staff workload during COVID-19 pandemic situations.

## Introduction

During the outbreak of rapidly transmitted infectious diseases, such as the novel Coronavirus disease 2019 (COVID-19) [1, 2], early isolation through the early detection of suspected patients is the most basic and important response strategy in emergency departments (EDs). Chest radiography (CR) constitutes a fast and relatively inexpensive imaging modality for finding lesions in the lungs. The portability of CR equipment can protect medical personnel from viruses and minimize the risk of spreading the virus [3, 4]. In this regard, Chung et al. [5], reported radiological abnormalities in 93.5% of the patients in EDs during the lockdowns through the pandemic that demonstrates the effectiveness of the clinical symptoms and CR-based revised triage and ED surveillance protocol. COVID-19 lung infection commonly produces ground-glass and consolidative opacities with a bilateral, peripheral, and lower lung distribution. Ground-glass opacity (GGO) lesions exhibit unclear boundaries, and the corresponding images are often not clear; this makes their detection in CRs difficult for non-expert clinicians. Real-time analysis by radiologists may improve the detection rate of COVID-19 pneumonia in CR; however, such analysis is impractical because of time and budgetary constraints.

The recent medical applications of deep learning (DL) algorithms have been attracting increasing attention. In particular, the performances of DL algorithms have attracted attention for the detection of pulmonary malignancy, active tuberculosis, pneumothorax, and pneumonia in CR images [6–9]. Previous research has demonstrated the clinical efficacy of the DL algorithm in terms of its ability to improve speed and accuracy in image reading [7, 10]. If the DL algorithm achieves a performance that is equivalent to that achieved by physicians in the detection of CR with COVID-19 pneumonia, the automatic interpretation of the CR with DL algorithms can significantly reduce the burden on clinicians and radiologists in a sudden surge of suspected COVID-19 patients. The aim of this study was to evaluate the efficacy of DL algorithms for detecting COVID-19 pneumonia on CR compared with radiological reports.

## Methods

### Study setting and population

By May 1, 2020, there were 6,852 confirmed COVID-19 patients in Daegu, Korea (the first case was detected on February 18, 2020) [11]. Daegu Metropolitan City has 15 emergency medical centers that provide health services to a population of 2.48 million and an area of 883.54 square kilometers. Of these, two level 1 EDs and three level 2 EDs participated in this study. These EDs provide the highest level of emergency care services in the region and annually care approximately 200,000 ED patients, accounting for 42.0% of patients visiting EDs in Daegu Metropolitan City [12]. Herein, we note that this study is a retrospective study of adult patients determined to be positive based on the COVID-19 reverse transcription polymerase chain reaction (RT-PCR) among all the patients who were admitted to five emergency medical centers and one community treatment center [13] in Daegu, Korea from February 18, 2020 to May 1, 2020. Of these, 12 patients who did not undergo CR were excluded from the study. The institutional review board (IRB) of Yeungnam University Medical Center reviewed and approved our study (IRB registration number: 2020-06-019). Subsequently, the IRBs of Daegu Catholic University Medical Center (CR-20-152), Daegu Fatima Hospital approved the study (DFE20ORIO073), Kyungpook National University Hospital (KNUH 2020-05-072), and Kyungpook National University Chilgok Hospital (KNUCH 2020-05-012). The requirement for the acquisition of informed consent was waived owing the retrospective nature of this study.

### Reference standards

The diagnosis of pneumonia in the present study was based on radiological findings. CR images without chest computed tomography (CT) scans were classified as positive for COVID-19 pneumonia if the radiologist identified GGO, consolidation, or other infiltration. Patients with evidence of pneumonia on chest CT scans were also classified as positive for COVID-19 pneumonia. In the case of CR images without CT scans, the results were re-evaluated by three thoracic radiologists for expert consensus [14]. The three radiologists independently determined whether patients had radiological evidence of pneumonia or not based on a retrospective review of the CRs. The final judgment was formulated based on the majority agreement of the three radiologists.

### Acquisition of CR images

Along with the relevant patient clinical data, the attending physician at each participating hospital used the Digital Imaging and Communications in Medicine standard to retrieve the anonymized initial CR images of COVID-19 patients who visited their ED. Each CR was matched with the formal reading report by a radiologist, and all the images were uploaded to a database after being anonymized. CR images exhibiting typical pneumonia symptoms, including GGO patterns, consolidation, or other infiltration, were classified as positive for pneumonia according to the formal reading report of hospital radiologists; otherwise, they were classified as negative.

### Analysis of CR images with DL algorithm

The CR images were evaluated using a commercialized DL algorithm capable of analyzing CR images (Lunit INSIGHT for CR 2, Lunit; accessible at https://insight.lunit.io) that was approved by the Ministry of Food and Drug Safety of Korea [7, 15]. The algorithm was developed to detect major thoracic diseases, including pulmonary malignancy, active pulmonary tuberculosis, pneumonia, and pneumothorax [7]. In our approach, upon the input of a CR image, classification predictions of the image are generated, and the neural network features of each slice of the CR image are combined by means of a max pooling operation. The resulting feature map is fed to a connected layer. The resulting output is a probability score for the class. The abnormality score of the DL algorithm reflects the likelihood of the presence of lesions. In the study, an abnormality score of >15% in which the indicated lesion location was consistent with the location of the actual lesion based on heatmap images was considered positive; whereas, abnormality scores >15% in which the indicated lesion location was not related to the actual lesion and abnormality scores <15% were defined as negative. In the study, the location of the lesion was confirmed by overlaying with the input radiograph. The evaluation of the localization accuracy was performed by a board-certified radiologist who reviewed all the heatmap images and determined if the DL algorithm was accurate in its classification. The classifications of the DL algorithm were considered correct when the lesion locations were accurate.

### Statistical analysis

The accuracy of the DL algorithm for the reference standard was calculated with a 95% confidence interval based on the DeLong method for the receiver operating characteristic (ROC) curve and area under the ROC curve (AUROC). Accordingly, the sensitivity, specificity, positive predictive values (PPV), and negative predictive values (NPV), were subsequently obtained. The sensitivities and specificities were compared using McNemar’s test, and the PPVs and NPVs were compared with the generalized score statistics. We also performed a subgroup analysis of COVID-19 patients who underwent chest CT scans. Statistical tests were performed with the use of SAS (version 9.4, SAS Institute Inc., Cary, NC, USA) and MedCalc (version 19.4.1, MedCalc Software, Ostend, Belgium). The results were considered to be statistically significant when the p-value was less than 0.05.

## Results

### Clinical characteristics of patients with COVID-19

**Fig 1:**
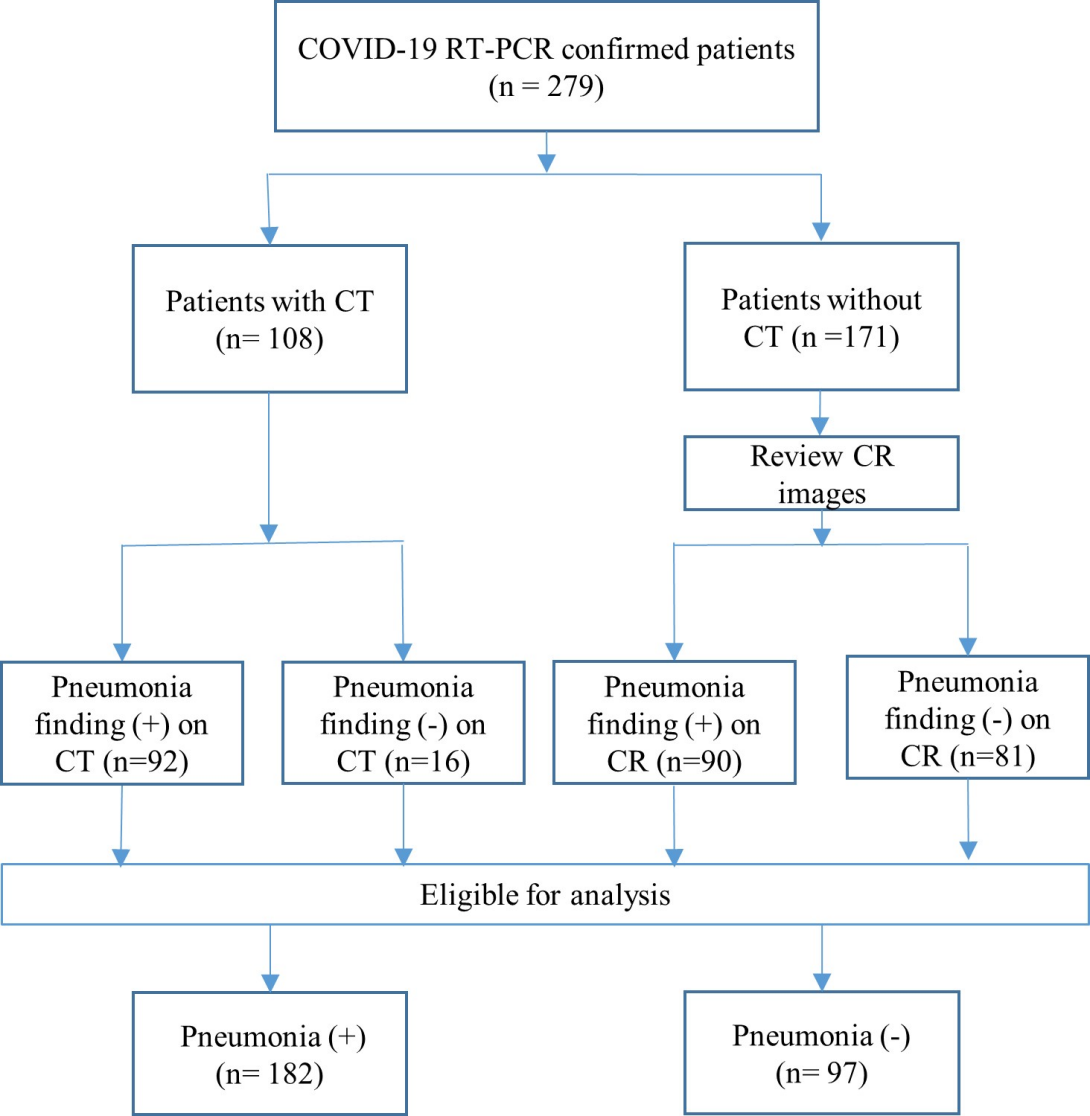
Flow chart of reference standard for COVID-19 with pneumonia and COVID-19 without pneumonia. CR, chest radiography; CT, computed tomography; RT-PCR, reverse transcription polymerase chain reaction.

In total, 279 patients were divided into two groups according to the interpretation of the CR images. The “COVID-19-with-pneumonia” group included 182 patients and the “COVID-19-without-pneumonia” group included 97 patients (Table 1). Among the 279 COVID-19 RT-PCR confirmed patients, 92 patients were diagnosed with COVID-19 with pneumonia, and 16 patients were diagnosed with COVID-19 without pneumonia as confirmed by chest CT scans (Fig 1). Of the 170 patients without CT scans, 91 patients were classified into the COVID-19-with-pneumonia group and 20 patients were classified into the COVID-19-without-pneumonia group based on the review of the CR images by a thoracic radiologist (Fig 1).

**Table 1:** Clinical characteristics of patients.

	Total	COVID 19 with pneumonia		COVID 19 without pneumonia		*P* value
	(N = 279)	(N = 182) (%)		(N = 97) (%)		
Sex						0.814
Male	135	89	48.9	46	47.4	
Female	144	93	51.1	51	52.6	
Age, years [IQR]	66 [44–79]	73.5 [64–81]		32 [26–58]		< .001
≥65	150	133	73.1	17	17.5	< .001
Signs and symptoms						
Fever	136	117	64.3	19	19.6	< .001
Cough	110	94	51.6	16	16.5	< .001
Sore throat	50	41	22.5	9	9.3	0.006
Sputum	69	50	27.5	19	19.6	0.146
Rhinorrhea	28	19	10.4	9	9.3	0.759
Myalgia	30	24	13.2	6	6.2	0.072
Diarrhea	16	16	8.8	0	0.0	0.003
Chest pain	9	8	4.4	1	1.0	0.169
Dyspnea	109	103	56.6	7	7.2	< .001
General weakness	33	28	15.4	4	4.1	0.005
Nausea	12	11	6.0	1	1.0	0.063
Abdominal pain	4	4	2.2	0	0.0	0.302
Headache	13	11	6.0	2	2.1	0.231
Dizziness	5	3	1.6	2	2.1	1.000
Asymptomatic	57	4	2.2	54	55.7	< .001
Institution						
A	38	36	19.8	2	2.0	< .001
B	36	28	15.4	8	8.2	
C	43	41	22.5	2	2.1	
D	63	50	27.5	13	13.4	
E	32	27	14.8	5	5.2	
F	67	0	0.0	67	69.1	
Chest radiography unit						
Standard	89	20	11.0	69	71.1	< .001
Bedside portable	190	162	89.0	28	28.9	
Time interval between symptom onset to chest radiography, Hr^a^ [IQR]	24.6 [7.0–76.8]	24.8 [6.8–85.2]		24.3 [9.3–71.6]		0.936

IQR, interquartile range.

^a^11 patients with unclear symptom onset and 57 asymptomatic patients were excluded.

The proportion of patients aged 65 years and older was 73.1% in the COVID-19-with-pneumonia group and 17.5% in the COVID-19-without-pneumonia group. In the COVID-19-with-pneumonia group, fever was the most common presenting symptom, followed by dyspnea, cough, and sputum. The numbers of asymptomatic patients were 4 (2.2%) and 54 (55.7%) in the COVID-19-with- and COVID-19-without-pneumonia groups, respectively.

### Performance of DL algorithm on chest radiograph images

**Fig 2:**
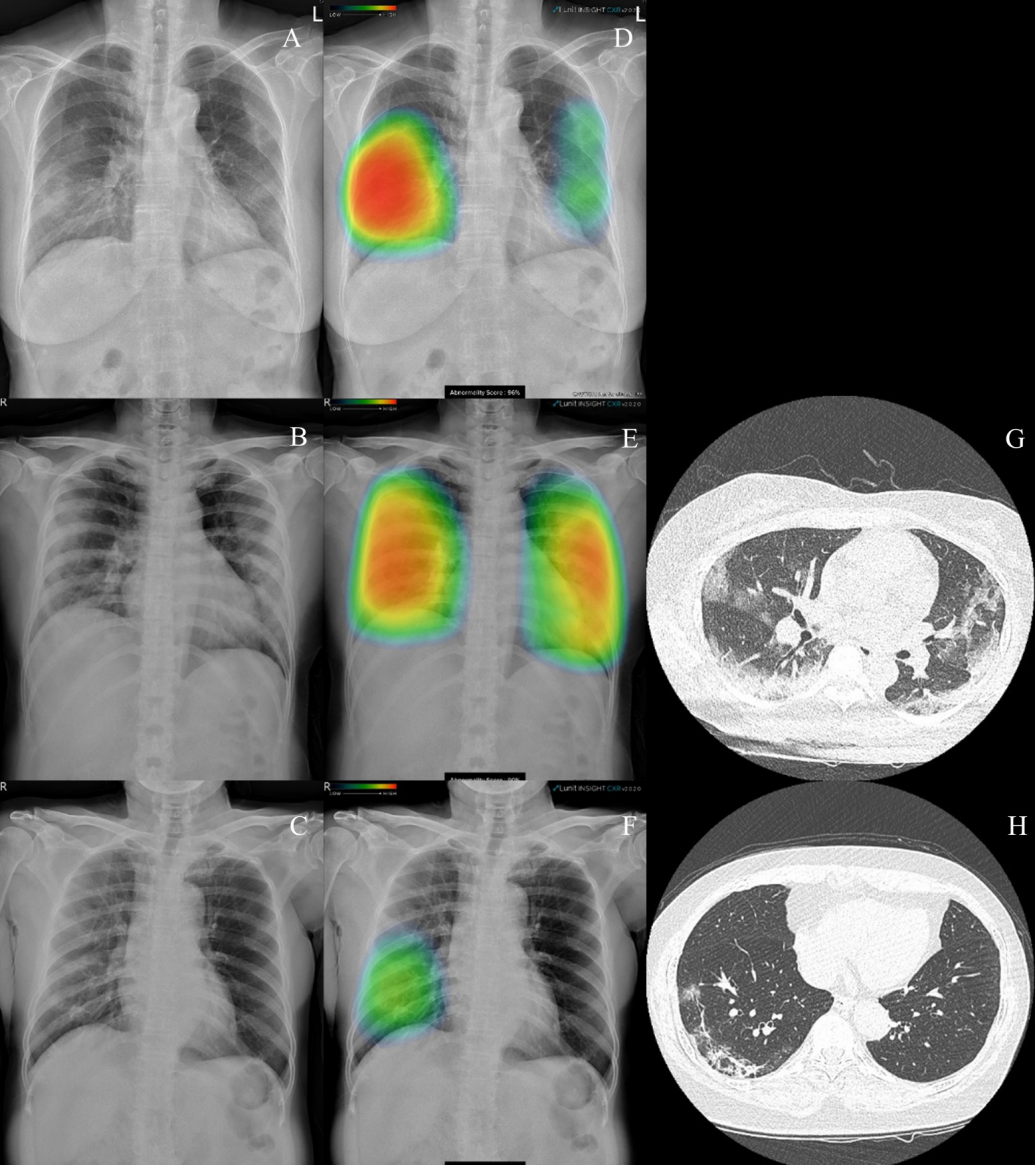
Results of the deep learning (DL) algorithm analysis of the localization of COVID-19 with pneumonia. (A, B, C) Chest radiography (D, E, F) DL algorithm heatmap overlaid on the image feature related to pneumonia. (G, H) Computed tomography scan, showing lung consolidation and ground-glass opacities that are suitably localized and detected by the DL algorithm.

The sensitivity and specificity of the DL algorithm were 95.6%, and 88.7%, respectively. No significant difference was observed in the AUROC value of the DL algorithm for the detection of COVID-19 with pneumonia compared with the radiology report (*P* = 0.322) (Table 2). Fig 2 shows the representative cases of CR and CT scans of the COVID-19-with-pneumonia group. Two of the illustrated cases show diffuse bilateral consolidation compatible with pneumonia. Another illustrated case shows right lower lung consolidation that was subsequently confirmed by CT scans. We note that each case is suitably localized and detected by the DL algorithm.

**Table 2:** Performance of deep learning algorithm for detection of COVID-19 with and without pneumonia on chest radiography.

	DL algorithm	Radiology report	*P* value
AUROC (95% CI)	0.921	0.941	0.322
	(0.883–0.950)	(0.906–0.965)	
Sensitivity (95% CI)	0.956	0.912	0.022
	(0.915–0.981)	(0.861–0.949)	
Specificity (95% CI)	0.887	0.969	0.039
	(0.806–0.942)	(0.912–0.994)	
PPV (95% CI)	0.941	0.982	0.023
	(0.901–0.965)	(0.948–0.994)	
NPV (95% CI)	0.915	0.855	0.022
	(0.845–0.955)	(0.786–0.904)	

AUROC, area under the receiver operating characteristic curve; CI, confidence interval; PPV, positive predictive values; NPV, negative predictive values; DL, deep-learning.

In subgroup analysis of patients on whom chest CT scans were performed, the sensitivity and specificity of the DL algorithm for detection of COVID-19 with and without pneumonia on CR were 74.9%, and 84.6%, respectively. The AUROC value of the DL algorithm and radiology report were 0.749 and 0.849, respectively (*P* = 0.258) (Table 3).

**Table 3:** Performance of deep learning algorithm for the detection of COVID-19 with and without pneumonia on chest radiography and in computed tomography performed on COVID-19 patients.

	DL algorithm	Radiology report	*P* value
AUROC (95% CI)	0.749	0.849	0.258
	(0.656–0.827)	(0.764–0.909)	
Sensitivity (95% CI)	0.935	0.880	0.125
	(0.863–0.976)	(0.796–0.939)	
Specificity (95% CI)	0.563	0.870	0.289
	(0.299–0.802)	(0.792–0.927)	
PPV (95% CI)	0.880	0.964	0.181
	(0.803–0.934)	(0.907–0.987)	
NPV (95% CI)	0.600	0.542	0.564
	(0.382–0.784	(0.393–0.683)	

AUROC, area under the receiver operating characteristic curve; CI, confidence interval; PPV, positive predictive values; NPV, negative predictive values; DL, deep-learning.

### False-positive interpretations by the DL algorithm

**Fig 3:**
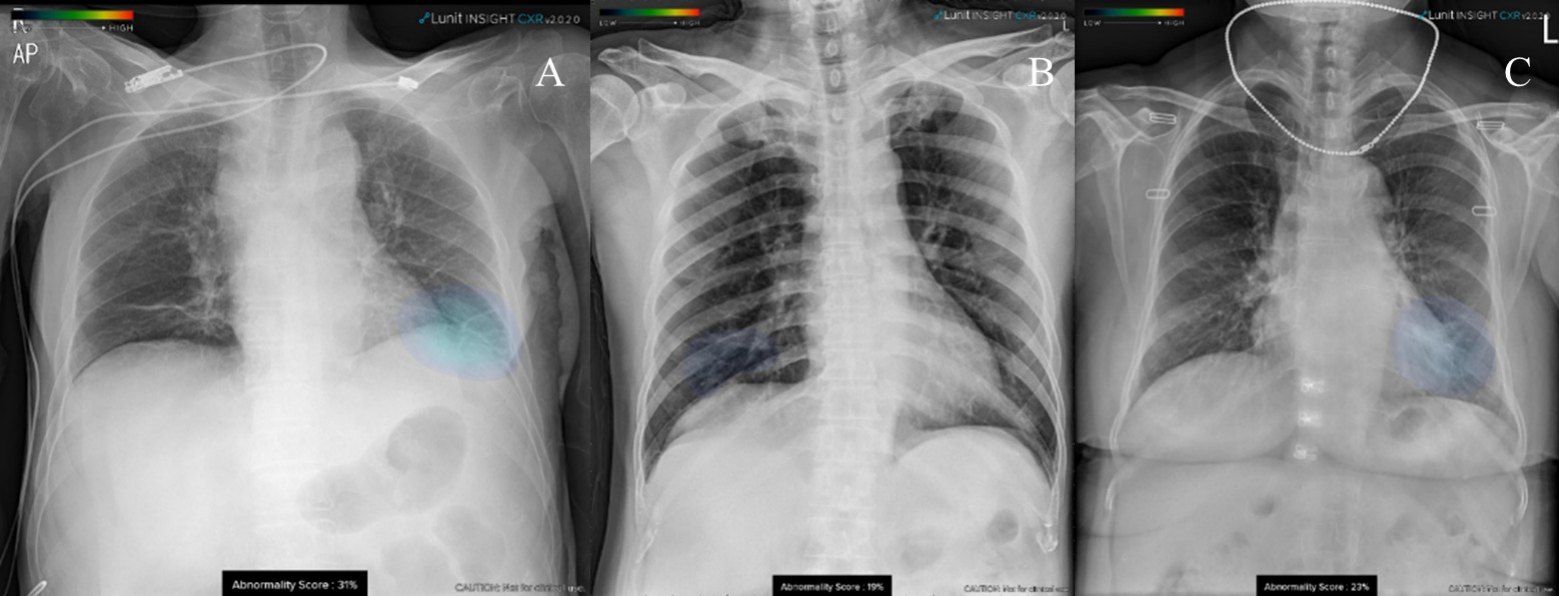
False-positive interpretations of DL algorithm. DL algorithm heatmap overlaid on image feature related to (A) interstitial thickening and (B, C) normal vascular marking.

There were 11 false-positive results obtained with the DL algorithm: normal vascular marking (n = 6), increased vascular marking (n = 2), emphysematous lung (n = 1), interstitial thickening (n = 1), and subsegmental atelectasis (n = 1). These results are shown in Fig 3.

### False-negative interpretations by the DL algorithm

**Fig 4:**
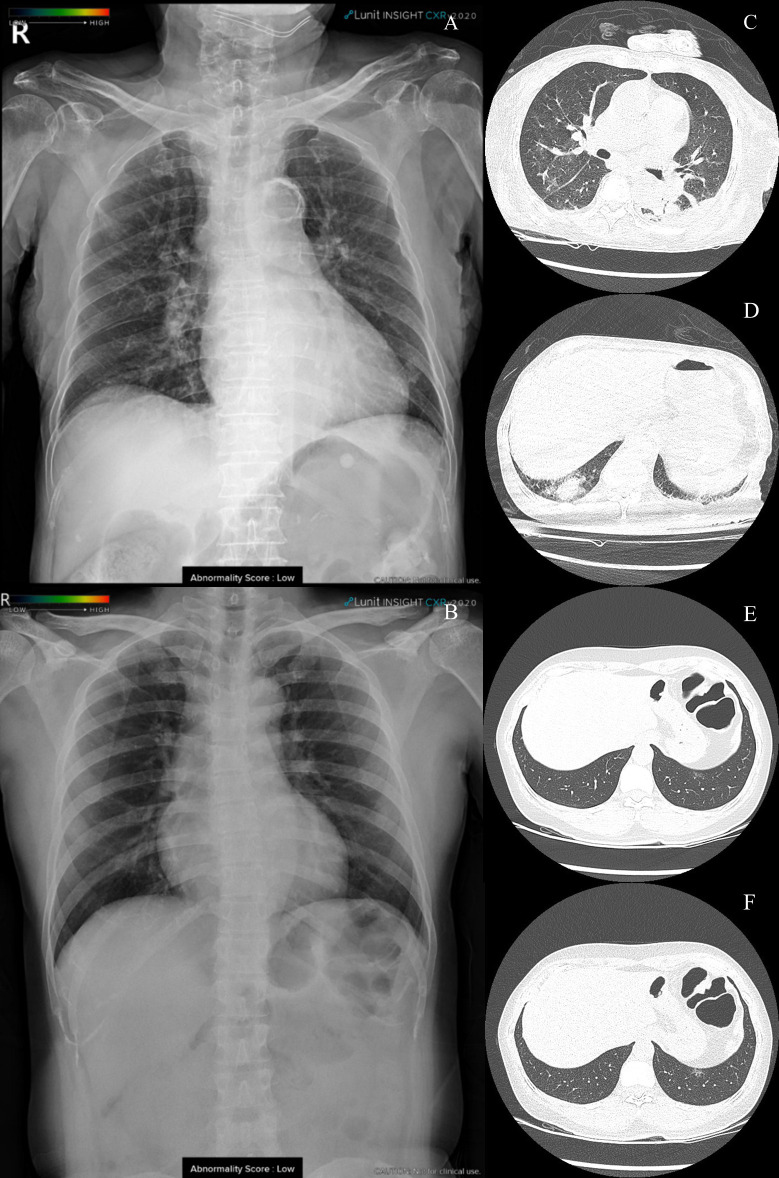
False-negative interpretations of DL algorithm. (A, B) DL algorithm classified chest radiography as negative for pneumonia. Chest computed tomography scans show (C) focal GGOs in the right middle lobe, (D) multifocal consolidations in the right lower lobe, and (E, F) small amounts of GGOs.

There were eight false-negative results obtained with the DL algorithm. One of the illustrated cases was interpreted as positive for pneumonia in the radiology report. A chest CT scan also showed small focal GGOs in the right middle lobe and multifocal consolidations in the right lower lobe. This case was reported as negative for pneumonia in both the radiology report and the DL algorithm. Another illustrated case shows a small amount of multifocal GGOs in chest CT scans (Fig 4).

## Discussion

The purpose of this study was to examine the performance of a DL algorithm that detects COVID-19 pneumonia from CR images compared with that of radiology reports. This study also validated the diagnostic performance and efficacy of a DL algorithm in analyzing the CR images of COVID-19 patients based on a multicenter study. The DL algorithm used in the study demonstrated a satisfactory diagnostic performance (AUROC = 0.921) in diagnosing pneumonia from the CR images of COVID-19 patients.

Regarding the detection of COVID-19 with pneumonia based on chest CT, both the DL algorithm and radiology reports yielded respective sensitivities of 93.5% and 88%. In this regard, Hurt et al. [16] reported that DL algorithms can have a higher applicable value for clinical diagnostic workflow based on their DL-algorithm-based CR analysis of five COVID-19 patients. According to previous studies performed in China [17, 18], chest CT yielded a high sensitivity of 97% and a low rate of 3.9% for missed diagnosis cases of COVID-19. Therefore, the sensitivity of the DL algorithm may be acceptable for use in clinical practice in COVID-19 pandemic situations.

February 18, 2020, demarcated the onset of the COVID-19 pandemic in Daegu, Korea. Since then, the median time of ED admissions to diagnosis of COVID-19 through RT-PCR was approximately 7 h in cases that resulted in ED temporary closure [5]. Additionally, the guidelines of the World Health Organization suggest the use of chest imaging for the diagnostic workup in symptomatic patients with suspected COVID-19 when RT-PCR testing is available but results are delayed [19]. Given that the timely diagnosis by RT-PCR can be limiting in a pandemic situation, and the fact that radiologic abnormality may precede the conclusive RT-PCR positive outcome, CR imaging may help the early diagnosis or triage of patients in pandemic situations. Moreover, considering the comparable diagnostic ability of the DL algorithm to that of CR for the detection of COVID-19 pneumonia, the observed clinical symptoms in conjunction with DL-based CR analysis may be useful in facilitating rapid decisions regarding in-hospital isolation, treatment facilities, or self-quarantine orders in EDs or screening clinics, particularly during pandemics.

In this study, there was a high proportion of relatively young asymptomatic patients in the COVID-19-without-pneumonia group. Approximately 7% of the asymptomatic patients were found to exhibit pneumonia as per CR, which clearly indicates that CR has limited value for diagnosing COVID-19 in asymptomatic patients. These results support the hypothesis that the efficacy of the image modality in the diagnosis can change depending on the outbreak and epidemic-phase status of nations [20].

There are several limitations to this study. First, this study was not a randomized, controlled study; thus, a potential for bias, which is characteristic of retrospective studies, may be present. Second, there was a difference in the period of time between the onset of symptoms and CR imaging [21]. Accordingly, the DL-algorithm-based CR results, clinical symptoms, and hemodynamic status should be considered in the classification of patients and their disposition. Third, there were 6,852 confirmed cases of COVID-19 in Daegu, but only 279 patients were included in the analysis. It is likely that a higher proportion of relatively severely ill patients who visited the EDs were included. Despite the fact that patients from the community treatment center who had mild symptoms or were asymptomatic were also included in the analysis, it is possible that there may have been selection bias. Fourth, the performance of a diagnostic test may vary depending on the characteristics of the target population. Although the algorithm yielded an acceptable performance in the present study, it may exhibit a lower performance in populations with a higher proportion of mild cases, or a lower prevalence. Fifth, this study comprised only confirmed COVID-19 patients and evaluated performance for the detection of pneumonia, not for the detection of COVID-19. Considering that a proportion of the patients did not exhibit pneumonia on CR, the performance of the DL algorithm for the identification of COVID-19 may be limited. Sixth, the successful use of CR in diagnosis depends on the different phases of the epidemic outbreak and the environments with varying critical resource availability [19, 20, 22]. Therefore, there may be limits to the generalization of the classification system based on the DL algorithm regarding its potential use in other countries.

## Conclusions

In this study, the DL algorithm demonstrated a satisfactory diagnostic performance comparable with that of radiology reports in the CR-based diagnosis of pneumonia in COVID-19 patients. In pandemic situations, such as the COVID-19, wherein medical resources and personnel are limited, the emergency medical system can be burdened considerably. In this context, the DL algorithm offers fast and reliable examinations that can facilitate decisions regarding patient screening and isolation, which can reduce the workload on medical staff.

## Supporting information

S1 Dataset De-identified participant dataset. (SAS7BDAT)
